# Clonal Expansion of Both Modern and Ancient Genotypes of *Mycobacterium tuberculosis* in Southern Taiwan

**DOI:** 10.1371/journal.pone.0043018

**Published:** 2012-08-24

**Authors:** Jia-Ru Chang, Yih-Yuan Chen, Tsi-Shu Huang, Wei-Feng Huang, Shu-Chen Kuo, Fan-Chen Tseng, Ih-Jen Su, Chien-Hsing Lin, Yao-Shen Chen, Jun-Ren Sun, Tzong-Shi Chiueh, Horng-Yunn Dou

**Affiliations:** 1 National Institute of Infectious Diseases and Vaccinology, National Health Research Institutes, Zhunan, Miaoli, Taiwan; 2 Institute of Molecular and Genomic Medicine , National Health Research Institutes, Zhunan, Miaoli, Taiwan; 3 Department of Microbiology, Kaohsiung Veterans General Hospital, Kaohsiung, Taiwan; 4 Division of Clinical Pathology, Department of Pathology, Tri-Service General Hospital and National Defense Medical Center, Taipei, Taiwan; 5 School of Medical Laboratory Science and Biotechnology, Taipei Medical University, Taipei, Taiwan; Institut de Pharmacologie et de Biologie Structurale, France

## Abstract

We present the first comprehensive analysis of *Mycobacterium tuberculosis* isolates circulating in the Kaohsiung region of southern Taiwan. The major spoligotypes found in the 224 isolates studied were Beijing lineages (n = 97; 43.3%), EAI lineages (n = 72; 32.1%) and Haarlem lineages (n = 18; 8.0%). By 24 MIRU-VNTR typing, 174 patterns were identified, including 24 clusters of 74 isolates and 150 unique patterns. The combination of spoligotyping and 12-MIRU-VNTR revealed that 129 (57.6%) of the 224 isolates were clustered in 18 genotypes. Moreover, 63.6% (7/11) of infected persons younger than 30 years had a Beijing strain, which could suggest recent spread among younger persons by this family of TB strains in Kaohsiung. Among the 94 Beijing family (SIT1, SIT250 and SIT1674) isolates further analyzed for SNPs by mass spectrometry, the most frequent strain found was ST10 (n = 49; 52%), followed by ST22 (n = 17; 18%) and ST19 (n = 11; 12%). Among the EAI-Manila family isolates analyzed by region deletion-based subtyping, the most frequent strain found was RD type 1 (n = 63; 87.5%), followed by RD type 2 (n = 9; 12.5%). In our previous study, the proportion of modern Beijing strains (52.5%) in northern Taiwan was significantly higher than the proportion of EAI strains (11%). In contrast, in the present study, EAI strains comprised up to 32% of Beijing strains in southern Taiwan. In conclusion, both ‘modern’ (Beijing) and ‘ancient’ (EAI) *M. tuberculosis* strains are prevalent in the Kaohsiung region, perhaps suggesting that both strains are somehow more adapted to southern Taiwan. It will be interesting to investigate the dynamics of the lineage composition by different selection pressures.

## Introduction

Tuberculosis (TB) remains a worldwide healthcare concern and is characterized by the World Health Organization (WHO) as an epidemic. TB is a leading notifiable communicable disease on the island of Taiwan. In 2008, 14,265 cases (62.0/100,000) were reported, with the highest numbers of new TB patients occurring in Taipei County (2,147 cases; 15.05%) and Taipei City (1,178; 8.25%) in the north and in Kaohsiung County (1,061; 7.43%) and Kaohsiung City (1,009; 7.07%) in the south. Kaohsiung is the second largest city of Taiwan, with a population of around 2.9 million. However, while the levels of TB infection and mortality are high in southern Taiwan, there are fewer documented genotyping studies of pulmonary *Mycobacterium tuberculosis* (MTB) isolates in southern Taiwan than in northern Taiwan [1]. Therefore, it is of interest to understand genotypic patterns of pulmonary MTB isolates in southern Taiwan and how they compare to those of northern Taiwan and elsewhere. Moreover, from a TB-control point of view, it is relevant to understand whether specific genotype families are over-represented among TB cases, potentially indicating higher rates of transmission of such MTB strains within the community. To that end we performed a hospital-based surveillance at a southern Taiwan medical center from 2006 to 2008, which involved characterizing the prevalence of genotypes and cluster patterns of MTB isolates in Kaohsiung City to obtain information on potential transmission and for formulation of infection-control policy.

## Materials and Methods

### Ethics statement

This study was approved by the Human Ethics Committee of the National Health Research Institutes, Taiwan (Code: EC0961103). Because of the retrospective nature, routine collection of clinical data in daily practice, and dislinkage of personal information, the requirement to obtain informed consent was waived by our institutional review board.

### Mycobacterial strains and genomic DNA

A total of 224 samples were collected between 2006 and 2008 from 224 patients at the Kaohsiung Veterans General Hospital, a large medical center that handles a substantial number of TB patients referred from hospitals throughout Kaohsiung. All of the patients were sputum microscopy positive and culture positive. Mycobacterial genomic DNA was extracted from cultured cells as described previously [2]. Briefly, mycobacterial colonies were resuspended in 100–200 µl of distilled H_2_O and incubated at 85°C for 30 min. After centrifugation of the suspension, the supernatant containing the DNA was removed and stored at −20°C until further use. The study protocol was approved by the institutional review board of the National Health Research Institutes, Taiwan.

### Spoligotyping and spoligotype analysis

Spoligotyping was carried out according to the manufacturer's instructions (Isogen Bioscience B.V., Maarsen, The Netherlands). The resulting spoligotypes were documented using a binary code representing either a positive or a negative hybridization result (n and o, respectively) and analyzed using Excel software for grouping and ordering of the patterns. The SITVITWEB database [3] and a web-based computer algorithm, Spotclust [4], were used to assign new isolates to families, subfamilies and variants. SITVITWEB assigned names (shared types) were used whenever a spoligopattern was found in the database. Patterns not found in SITVITWEB were assigned to families and subfamilies by Spotclust. Spoligotypes described only once (non-clustered) in this study and in the SITVITWEB were designated as “orphan”. A cluster was defined as two or more isolates from different patients with identical spoligotype patterns.

### PCR and MIRU analysis

PCRs were carried out using a PCR reagent system (Gibco-BRL). Sequences of the primers used for amplification of the 24 MIRU loci were selected according to descriptions in other studies [5]. Five microliters from fivefold-diluted DNA solutions were added to a final volume of 50 µl containing 0.2 µl of DNA polymerase (1 U); 0.2 mM each of dATP, dCTP, dGTP, and dTTP; 5 µl of PCR buffer; 0.4 µM (2 µM for locus 7) of primers; and 1 to 3.5 mM of MgCl_2_. The primers and MgCl_2_ concentrations used were as described by Mazars *et al.* [6]. The PCR fragments were analyzed by 2% agarose gel electrophoresis (Figure S1). The sizes of the amplicons were estimated by comparison with 50- and 100-bp ladders. The MIRU copy number per locus was calculated by using the conventions described by Supply *et al.* [7].

### Single-nucleotide polymorphism typing

PCR and extension primers were designed using MassArray Assay Design 3.1 software (Sequenom, San Diego, CA). PCRs contained, in a volume of 5 µL, 1 pmol of the corresponding primers, 10 ng genomic DNA, and HotStar reaction mix (Qiagen) in 384-well plates. PCR conditions were as follows: 94°C for 15 min, followed by 40 cycles of 94°C (20 s), 56°C (30 s), 72°C (60 s), and a final extension of 72°C for 3 min. In the primer extension procedure, each sample was denatured at 94°C, followed by 40 cycles of 94°C (5 s), 52°C (5 s), 72°C (5 s). The mass spectrum from time-resolved spectra was retrieved by using a MassARRAY mass spectrometer (Sequenom), and each spectrum was then analyzed using SpectroTYPER software (Sequenom) to perform the genotype calling. A detailed explanation of this methodology has been described [8].

### Detection of RD deletions

EAI family strains in our collection were further classified by using PCR amplification to determine the RD deletion genotype. A primer set was used to check for the presence or absence of RD131ab, RD135, RD141, RD147c, and RD239. The PCR mixture consisted of 0.2 µg DNA template, 13.9 µl Q buffer, 5 µl 5× buffer, 4 µl 10 mM deoxynucleoside triphosphates, 1 µl of each primer (10 pmol/µl), 1 µl DMSO, and 0.6 µl Herculase II Fusion DNA polymerase (Stratagene, USA). Sterile water was used to dilute the mixture up to 25 µl. Regions amplified by PCR were sequenced to confirm the reaction. A detailed explanation of this methodology has been described [9].

### Drug resistance testing

The proportional method for drug susceptibility testing (DST) of MTB was performed as described previously [10]. Briefly, for each drug a 1∶10 dilution of standardized suspension was inoculated onto the control and drug-containing media. The extent of growth in the absence or presence of the drug was compared and expressed as a percentage. If growth at the critical concentration of a drug was >1%, the isolate was considered to be clinically resistant. 7H10 agar with 0.2 or 1 mg/L isoniazid (INH), 1 or 5 mg/L rifampicin, 5 or 10 mg/L ethambutol, and 5 or 10 mg/L streptomycin was used.

### Statistical analysis

The Hunter–Gaston equation [11], an application of Simpson's index of diversity [12], was used to calculate the allelic diversity, or the ‘Hunter–Gaston discrimination index’ (HGDI), at each locus. The equation is:
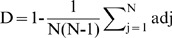
where *D* is the index of discriminatory power, *a_j_* is the number of strains in the population which are indistinguishable from the *j*th strain, and *N* is the number of strains in the population [13].

## Results

### Analysis of spoligotyping patterns

A total of 224 isolates from 224 patients (median age = 68 years; 72.8%, n = 163 were male) who were diagnosed with culture-confirmed TB were subjected to spoligotyping and MIRU-VNTR typing (Table S1). Molecular analysis showed that all of the TB cases were caused by *M. tuberculosis*, except one by *Mycobacterium bovis*. Of the 224 isolates analyzed, spoligotypes from 209 isolates (93.3%) were classified according to SITVITWEB into one of 33 shared international types (SITs) (Table 1). Of these defined spoligotypes the most frequent strains found were Beijing SIT1 (40.6%) and East African-India (EAI)_2 Manila SIT19 (27.7%) (Table 1). Of the remaining 15 isolates that were not represented in SITVITWEB, 2 were EAI, 5 were Family-33, 4 were Haarlem, 1 was T, 1 was LAM, 1 was Family-36, and 1 was Beijing, based on Spotclust (Table 2).

**Table 1:** Spoligotypes of 209 isolates with a shared international type (SIT) number in SITVITWEB database.

SIT^a^	Spoligotype	Label^b^	No. of isolates^c^	Prevalence^d^ (%)
**1**	□□□□□□□□□□□□□□□□□□□□□□□□□□□□□□□□□□▪▪▪▪▪▪▪▪▪	**Beijing**	**91**	**40.63**
**250**	□□□□□□□□□□□□□□□□□□□□□□□□□□□□□□□□□□□□□▪▪▪▪▪▪	**Beijing**	**1**	**0.45**
**1311**	□□□□□□□□□□□□□□□□□□□□□□□□□□□□□□□□□□▪▪▪▪▪□□□□	**Beijing**	**3**	**1.34**
**1674**	□□□□□□□□□□□□□□□□□□□□□□□□□□□□□□□□□□▪▪▪▪▪▪▪□▪	**Beijing**	**1**	**0.45**
**36**	▪▪▪▪▪▪▪▪▪▪▪▪□▪▪▪▪▪▪▪▪▪▪▪▪▪▪▪▪▪□▪□□□□▪▪▪▪▪▪▪	**H3**	**1**	**0.45**
**50**	▪▪▪▪▪▪▪▪▪▪▪▪▪▪▪▪▪▪▪▪▪▪▪▪▪▪▪▪▪▪□▪□□□□▪▪▪▪▪▪▪	**H3**	**6**	**2.68**
**99**	▪▪▪▪□▪▪▪▪▪▪▪▪▪▪▪▪▪▪▪▪▪▪▪▪▪▪▪▪▪□▪□□□□▪▪▪▪▪▪▪	**H3**	**1**	**0.45**
**742**	▪▪▪▪▪▪▪▪▪▪▪▪▪▪▪▪▪▪▪▪▪▪▪▪□□□□□□□▪□□□□▪▪▪▪▪▪▪	**H**	**3**	**1.34**
**946**	▪▪▪▪▪▪▪▪▪▪▪▪▪▪▪▪▪▪▪▪▪▪□□□□□□□□□▪□□□□▪▪▪▪▪▪▪	**H**	**2**	**0.89**
**2092**	▪▪▪▪▪▪▪▪▪▪▪▪□▪▪▪▪▪▪▪▪▪▪▪□□□□□□□▪□□□□▪▪▪▪▪▪▪	**H3**	**1**	**0.45**
**53**	▪▪▪▪▪▪▪▪▪▪▪▪▪▪▪▪▪▪▪▪▪▪▪▪▪▪▪▪▪▪▪▪□□□□▪▪▪▪▪▪▪	**T1**	**6**	**2.68**
**131**	▪▪▪▪▪▪▪▪▪▪▪▪□□▪▪▪▪▪▪▪▪▪▪▪▪▪▪▪▪▪▪□□□□▪▪▪▪▪▪▪	**T1**	**1**	**0.45**
**249**	▪▪▪▪▪▪▪▪▪▪▪□▪□□□□□□□□□□□▪▪▪▪▪▪▪▪□□□□▪▪▪▪▪▪▪	**LAM**	**3**	**1.34**
**917**	▪▪▪▪▪▪▪▪▪▪□▪▪▪▪▪▪▪▪▪▪▪▪▪▪▪▪▪▪▪▪▪□□□□▪▪▪▪▪▪▪	**T1**	**1**	**0.45**
**926**	▪▪▪▪▪▪□▪▪▪▪▪▪▪▪▪▪▪▪▪▪▪▪▪▪▪▪▪▪▪▪▪□□□□▪▪▪▪▪▪▪	**T1**	**1**	**0.45**
**2393**	▪▪▪▪▪▪▪▪▪□□□□▪▪▪▪▪▪▪▪▪▪▪▪▪▪▪▪▪▪▪□□□□▪▪▪▪▪▪▪	**T1**	**2**	**0.89**
**52**	▪▪▪▪▪▪▪▪▪▪▪▪▪▪▪▪▪▪▪▪▪▪▪▪▪▪▪▪▪▪▪▪□□□□▪▪▪□▪▪▪	**T2**	**2**	**0.89**
**848**	▪▪▪□▪▪▪▪▪▪▪▪▪▪▪▪▪▪▪▪▪▪▪▪▪▪▪▪▪▪▪▪□□□□▪▪▪□▪▪▪	**T2**	**1**	**0.45**
**1302**	▪□▪▪▪▪▪▪▪▪▪▪▪▪▪▪▪▪▪▪▪▪▪▪▪▪▪▪▪▪▪▪□□□□▪▪▪□▪▪▪	**T2**	**1**	**0.45**
**19**	▪▪□▪▪▪▪▪▪▪▪▪▪▪▪▪▪▪▪□□▪▪▪▪▪▪▪□□□□▪□▪▪▪▪▪▪▪▪▪	**EAI2_Manila**	**62**	**27.68**
**89**	▪▪□▪▪▪▪□□□□□□□□□□□□□□□□□□▪▪▪□□□□▪□▪▪▪▪▪▪▪▪▪	**EAI_Nonthaburi**	**1**	**0.45**
**483**	▪▪□▪▪▪▪▪▪▪▪▪▪▪▪▪▪▪▪□□▪▪▪▪▪▪▪□□□□▪□▪▪▪▪▪□□□▪	**EAI2_Manila**	**1**	**0.45**
**894**	▪▪□▪▪▪▪▪▪▪▪▪▪▪▪▪▪▪▪□□▪▪▪▪▪▪▪□□□□▪□▪▪▪▪▪□▪▪▪	**EAI2_Manila**	**1**	**0.45**
**2085**	▪▪□▪▪▪▪□□□▪▪▪▪▪▪▪▪▪□□▪▪▪▪▪▪▪□□□□▪□▪▪▪▪▪▪▪▪▪	**EAI2_Manila**	**1**	**0.45**
**2098**	▪▪□▪□□▪▪▪▪▪▪▪▪▪▪▪▪▪□□▪▪▪▪▪▪▪□□□□▪□▪▪▪▪▪□▪▪▪	**EAI2_Manila**	**1**	**0.45**
**2351**	▪▪□▪□▪▪▪▪▪▪▪▪▪▪▪▪▪▪□□▪▪▪▪▪▪▪□□□□▪□▪▪▪▪▪▪▪▪▪	**EAI2_Manila**	**1**	**0.45**
**2097**	▪▪□□□▪▪▪▪▪▪▪▪▪▪▪▪▪▪□□▪▪▪▪▪▪▪□□□□▪□▪▪▪▪▪▪▪▪▪	**EAI5**	**2**	**0.89**
**177**	□▪▪▪▪▪▪▪▪▪▪▪▪▪▪▪▪▪▪▪□□□□▪▪▪▪▪▪▪▪□□□□▪▪▪▪▪▪▪	**LAM9**	**2**	**0.89**
**246**	▪▪▪▪▪▪▪▪▪▪▪▪▪▪▪▪▪▪▪▪▪▪▪▪▪▪▪▪▪▪▪▪▪▪▪▪▪▪▪□▪▪▪	**undefined** e	**2**	**0.89**
**523**	▪▪▪▪▪▪▪▪▪▪▪▪▪▪▪▪▪▪▪▪▪▪▪▪▪▪▪▪▪▪▪▪▪▪▪▪▪▪▪▪▪▪▪	**MANU-ancestor**	**3**	**1.34**
**574**	▪▪▪▪▪▪▪▪▪▪▪▪▪▪▪▪▪▪▪▪▪▪▪▪▪▪▪▪▪▪▪□□□□□□□□▪▪▪▪	**T1**	**1**	**0.45**
**2587**	▪▪▪▪▪▪▪▪▪▪▪▪□▪▪▪▪▪▪▪▪▪▪▪▪▪▪▪▪▪▪▪▪▪▪▪▪▪▪□▪▪▪	**undefined** e	**2**	**0.89**
**684**	▪▪□▪▪□▪▪□▪▪▪▪▪▪□▪▪▪▪□▪▪▪▪▪▪▪▪▪▪▪▪▪▪▪▪▪□□□□□	**BOV_1**	**1**	**0.45**

a Shared international type (SIT), international spoligotype database SITVITWEB (**SITVITWEB, database.** http://www.pasteur-guadeloupe.fr:8081/SITVIT_ONLINE).

b Label representing spoligotype families as assigned in the international spoligotype database SITVITWEB.

c Number of isolates in this study.

d Prevalence, representing the number of isolates with a common SIT relative to the total number of isolates from the same database (224) classified by SIT from Kaohsiung Veterans General Hospital (expressed as a percentile).

e Undefined in SITVITWEB database.

**Table 2:** Spoligotypes of 15 orphan strains identified in Spotclust.

Spoligotype	MIRU-VNTR typing	No. of isolates	Spotclust	Prevalence (%)
000000000000171	**4242233251735334544336112**	**1**	**Beijing** a	**0.45**
577777603720771	**314222325153323224233282**	**1**	**LAM**	**0.45**
003777477413771	**464254326223432193232951**	**1**	**EAI** b	**0.45**
657777477413731	**4642543262234321103232951**	**1**	**EAI** b	**0.45**
001777770020771	**314222325153323234233382**	**1**	**H1**	**0.45**
777777570000731	**534228225173433544231981**	**1**	**H1**	**0.45**
777577775720771	**314222325153323134233352**	**2**	**H3**	**0.89**
774000170020771	**314222325153323234233392**	**1**	**T3**	**0.45**
777617777777761	**524227225173423444231861**	**1**	**Family33**	**0.89**
	**524228225173424444231861**	**1**		
777777400037771	**534227225153433444231881**	**1**	**Family33**	**0.45**
777777557777771	**524225225173433444221881**	**1**	**Family33**	**0.45**
777777767777771	**534226225173433444231881**	**1**	**Family33**	**0.45**
700000007760731	**424223325735334544336112**	**1**	**Family36**	**0.45**

a : RD105 deletion.

b : TBD1 positive.

Overall, the Beijing family was the most prevalent genotype, identified in 97/224 (43.3%) isolates, followed by the EAI family, identified in 72/224 (32.1%) isolates (Table 3). Among the 15 novel spoligotypes, 2 strains were found to be TBD1 positive and to lack DR spacers 29 to 32 and 34 but to possess spacer 33. Based on this result, these 2 new spoligotypes belong to the EAI family.

**Table 3:** Comparative analysis of the genotyping methods applied to study the clinical *Mycobacterium tuberculosis* strains circulating in Kaohsiung in southern Taiwan.

Typing methods, Strain group	Number of genotypes in total	No. of unique genotypes	No. of clusters	No. of strains in clusters	HGDI value
**Spoligotyping**					
**All strains**	46	29	17	2-91	0.758
**Beijing strain only**	5	3	2	3-91	0.063
**Non-Beijing strains**	41	26	15	2-62	0.768
**VNTR analysis, all strains**					
**12-MIRU-VNTR**	91	69	22	2-52	0.917
**24-MIRU-VNTR**	174	150	24	2-10	0.995
**Spoligotyping+12-MIRU**					
**All strains**	113	95	18	2-43	0.937
**Beijing strains only**	42	33	9	3-35	0.863
**Spoligotyping+24-MIRU**					
**All strains**	181	159	22	2-9	0.996
**Beijing strains only**	80	72	8	2-5	0.994

### Comparative analysis of genotyping methods

We calculated the HGDI value, a measure of allelic diversity, for each of the different genotyping methods. For the Beijing strains, the HGDI value was 0.063 for spoligotyping and 0.863 for the 12-MIRU loci set. The 24-VNTR loci scheme demonstrated the highest discriminatory power (0.995) for a single test. The best discriminatory power was demonstrated by a combination of 24 loci VNTR analysis and spoligotyping: the HGDI value was 0.996 for all strains and was 0.994 for Beijing strains only (Table 3).

### Characteristics of the TB patients

**Figure 1:**
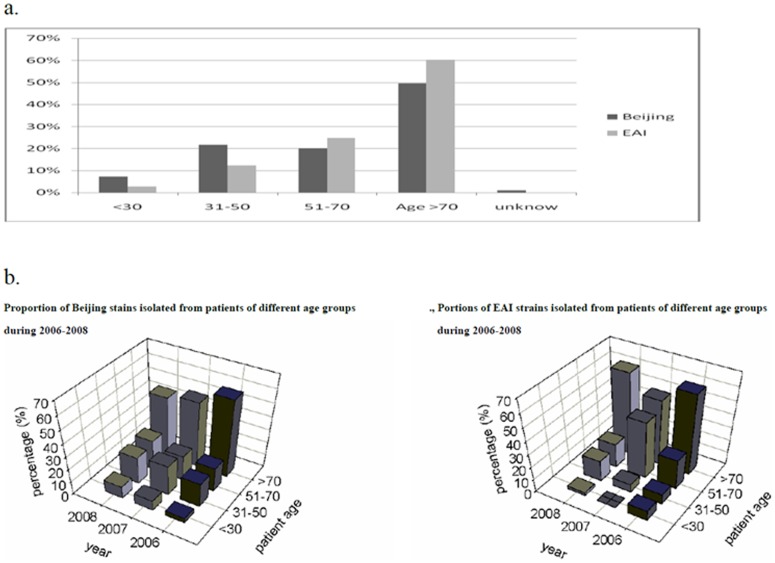
Patient age versus distribution of Beijing and EAI family isolates from 224 tuberculosis cases in Kaohsiung, 2006–2008. (a) Age distribution of TB patients (b) Trends in the percentage of Beijing and EAI MTB genotypes.

The average age (in years) of patients infected with each of the MTB genotypes was as follows: Beijing: 66; EAI: 69.7; Haarlem: 62.7; T: 67; Family-33: 70.4; LAM: 72.8 and Manu_ancestor: 79. The composition of Beijing and EAI lineages in young TB patients was very different compared with that in older ones (Table 4 and Figure 1). The prevalence of Beijing strains decreased as the age of patients increased, whereas that of EAI gradually increased (Table 4). The prevalence of Beijing strains in different age groups (age ≤30, 31–50, 51–70 and >70) was 63.6%, 57.9%, 37.0%, and 41.0%, respectively (p = 0.053) whereas that of EAI was 18.2%, 23.7%, 33.3%, and 36.8%, respectively (p = 0.077). Furthermore, the age distributions across sampling years were compared within each genotype: the percentage of Beijing-infected patients ≤30 years old increased monotonically from 3.9% in 2006 to 9.3% in 2008, but no such increasing trend was observed for all non-Beijing genotypes alone or combined (Figure 1). Thus, the balance that normally exists between the proportion of Beijing and EAI lineages in older patients appears to be in flux in young TB patients (Figure 1). The MTB-infected young population is increasingly more likely to be infected with a Beijing strain than with any other strain as compared to the older population, which may suggest a possible recent spread of the Beijing genotype in young persons in Kaohsiung.

**Table 4:** Characteristics of the tuberculosis (TB) patients from whom the study's *Mycobacterium tuberculosis* strains were isolated.

Genotype family	Beijing*	EAI	Haarlem	T	Family33	Family36	LAM	Manu_ancestor	Bovis
**No. of isolates**	97	72	18	17	5	1	6	3	1
**(%)**	(43.3)	(32.1)	(8.0)	(7.6)	(2.2)	(0.4)	(2.7)	(1.3)	(0. 4)
**Sex**									
**male**	74	52	11	14	3	1	5	1	1
**female**	23	20	7	5	2	0	1	2	0
**Age (mean)**	66	69.7	62.7	67	70.4	81	72.8	79	36
**(median)**	70	73.5	63.5	73	65		80.5	77	
**≤30**	7	2	1	1	0	0	0	0	0
**31–50**	22	9	4	1	0	0	1	0	1
**51–70**	20	18	6	6	3	0	1	0	0
**>70**	48	43	7	9	2	1	4	3	0

### Subtyping of Beijing and EAI strains

From a collection of 97 Beijing strains, a total of 94 Beijing sublineages were identified by SNPs. The most frequent strain found was ST10 (52%), followed by ST22 (18%) and ST19 (12%), which all belong to modern sublineages. An original sample set of 72 EAI family isolates was further analyzed by RD typing. The most common typing pattern, type 1, without deletions, was shared by 63 of the 72 isolates (Table 5).

**Table 5:** The most common typing patterns of 5 RD deletion regions seen in Manila family isolates.

Type	Pattern^a^	No. of isolates
1	+++++	63
2	+−+++	9
Total		72

a Symbols indicate the presence (+) or deletion (−) of the following regions of difference, in order: RD131ab, RD135, RD141, RD147c, and RD239.

### Drug susceptibility testing

Among the 224 strains in this study, 175 isolates (78.1%) were sensitive to all four of the first-line agents tested, 44 (19.6%) were resistant to at least one drug, and 5 (2.2%) were multidrug resistant (MDR) (Table 6). Analysis of the association between MDR and genotypes (as determined by spoligotyping) showed that 4 MDR strains were of the Beijing genotype.

**Table 6:** Mycobacterial genotype and drug resistance in patients with culture-confirmed tuberculosis.

Drug susceptibility results (no of isolates)				
Genotype family	No. of isolates	MDR (%)	Any one drug (%)	Sensitive to all drugs (%)
Beijing^b^	97(43.3%)	4 (4.1)	20 (20.6)	72 (74.2)
EAI	72 (32.1%)	0	12 (16.7)	60 (83.3)
T	17 (7.6%)	0	4 (23.5)	13 (88.2)
Haarlem	18 (8.0%)	0	2 (11.1)	16 (88.9)
Others^a^	20(8.9%)	1(5)	6 (30)	13 (65)
Total	224	5 (2.2)	44 (19.6)	175 (78.1)

a ‘Others’, all genotype families with a frequency of less than 10 cases **(LAM,, MANU-ancestor, Family-33, Family36 and Bovis)**.

b 3 ST1311(U lineage) isolates belong to the Beijing family.

## Discussion

The present study revealed Beijing strains to be the dominant TB pathogen in Kaohsiung in southern Taiwan (43.3% of cases), which is a lower percentage than what we recently observed in northern Taiwan (52.5% of cases) (p = 0.0377) [1]. A similar distribution trend was also recently observed by Huang *et al.* (Beijing strains: North, 53%; South, 27%, EAI strains: South, 20%) [14]. As its name implies and has generally has been accepted, the Beijing genotype originated in the Beijing area in the north of China [15], [16] although a recent study suggested its hypothetical origin to be in the Guangxi province in the south of China [17], and strains of this family were found to be dominant not only in China (82%), but also in neighboring countries such as Indonesia (44%), Thailand (44%), Vietnam (53%), Japan (79%), and South Korea (91%) [16]–[21]. Our recent report of MTB isolates in northern Taiwan showed the most frequent Beijing sublineage there to be ST10 (53.3%), followed by ST19 (14.8%) and ST22 (14.5%) [8]; in the present study, we found a similar distribution of Beijing sublineages in Kaohsiung. In contrast to the distribution of modern Beijing family strains seen in Taiwan and other countries worldwide, the ancient Beijing strains are dominant in Japan [8], [19], [22]. We believe that the modern Beijing strains, with their high degree of transmissibility, are currently spreading throughout the world. It was previously reported that BCG vaccination favors the positive selection of modern Beijing strains [23]. Our results are consistent with this assertion.

Nearly 7% (15/224) of the spoligotypes characterized in the present study were absent from the SITVITWEB database, which suggests that a large number of genotypes prevalent in southern Taiwan is still not identified. Further analysis by Spotclust revealed the majority of these to be Haarlem strains and Family-33 strains.

The EAI lineage, the most ancient MTB lineage, was first described in Guinea-Bissau [24] and predominates in Southeast Asia, particularly in the Philippines (73%), in Myanmar and Malaysia (53%), and in Vietnam and Thailand (32%) [3]. After modern humans dispersed out of Africa about 60,000 years ago [25] they migrated to the Asian mainland aided by low sea levels during the last ice age [26]. Subsequent prehistoric migrations to islands of East Asia and the Pacific have been designated differently depending on whether they were traced by language, archeological remains, or genetic studies. Most of the native Pacific languages from near the African coast through to Polynesia are Malayo-Polynesian, a subgroup of the Austronesian language family [27]. The nine other subgroups of Austronesian are spoken only in Taiwan, suggesting that Taiwan is the origin of Austronesian [28]. The high prevalence of the EAI lineage in Southeast Asia and southern Taiwan, areas which also share related Austronesian languages, suggests a possible relationship between EAI spreading and migration of Austronesian-speaking peoples, but this hypothesis needs further investigation. EAI strains in northern Taiwan comprise about 11% of MTB isolates, but in southern Taiwan they comprise up to 32%. It is very interesting that, despite the same ethnic background, southern Taiwan has a higher proportion of EAI than northern Taiwan. One possible explanation for this difference could be the more tropical climate of southern Taiwan, which is similar to that of the Philippines and Malaysia. Perhaps EAI strains are better adapted for growth and transmission in high-temperature environments, but this remains to be determined. EAI is characterized by a low copy number of IS6110, absence of spacers 29–32 and 34 and presence of spacer 33 (by spoligotyping), presence of the TBD1 region [29], and the RD239 deletion [29].

Fitness is a genotype-by-environment interaction. *M. tuberculosis* lives in a complex environment within the human host where numerous adaptive pressures are applied. Both modern (Beijing) and ancestral (EAI) *M. tuberculosis* strains are prevalent in southern Taiwan, suggesting that both types are somehow adapted to the region. Reasons for the successful adaption of the EAI lineage in a given geographical area have to be explored by studying host–pathogen interactions and immune responses. It will be interesting to investigate the dynamics of lineage composition under different selection pressures. The apparent changing proportions of modern and ancient genotypes of *M. tuberculosis* in young TB patients in southern Taiwan might be because of natural selection, possibly skewed by the two major health-measures against tuberculosis: BCG vaccination and antituberculosis treatment [30].

The aim of this study was to acquire data that can be used to enhance TB control activities through identification of previously unrecognized chains of transmission and by monitoring disease trends, including drug resistance phenotypes. Knowledge of these factors will enable more precise allocation of public health resources. Strain analysis, together with virulence studies, will also help in pinpointing isolates associated with higher morbidity and mortality, with the aim of directing efforts to limit the spread of those strains within the region. This study gives the first overview of the *M. tuberculosis* strains circulating in southern Taiwan. Based on a combination of spoligotyping and MIRU-VNTR, our data show that the Beijing strain has a high number of clusters in our sample population. The high prevalence of the Beijing genotype in the younger segment of the population warrants close attention to control policy and vaccination strategy.

In conclusion, Beijing and EAI are the prevalent lineages in Kaohsiung in southern Taiwan. One can assume that TB in this geographic region is shaped by the clonal expansion of both modern and ancient TB strains. The apparent changing proportions of modern and ancient genotypes of *M. tuberculosis* in young TB patients of Kaohsiung might be because of BCG vaccine selection. In spite of the presence of highly transmissible lineages like Beijing, there is no significant association with drug resistance in any of the lineages.

## Supporting Information

Figure S1 The quality test of MIRU-VNTR typing. Three loci was amplified and analyzed by electrophoresis using a 2% agarose gel. M, size markers; C, H37Rv control; lane 1–20; clinical isolates.
(TIF)

Table S1 24-MIRU-VNTR, spoligotyping and drug resistant profiles of 224 clinical isloates in this study.
(XLS)
