# Supplementary figures and images for: Clonal Expansion of Both Modern and Ancient Genotypes of Mycobacterium tuberculosis in Southern Taiwan
Source: PLoS One. 2012 Aug 24;7(8):e43018. doi: 10.1371/journal.pone.0043018 (PMC3427295; doi:10.1371/journal.pone.0043018)

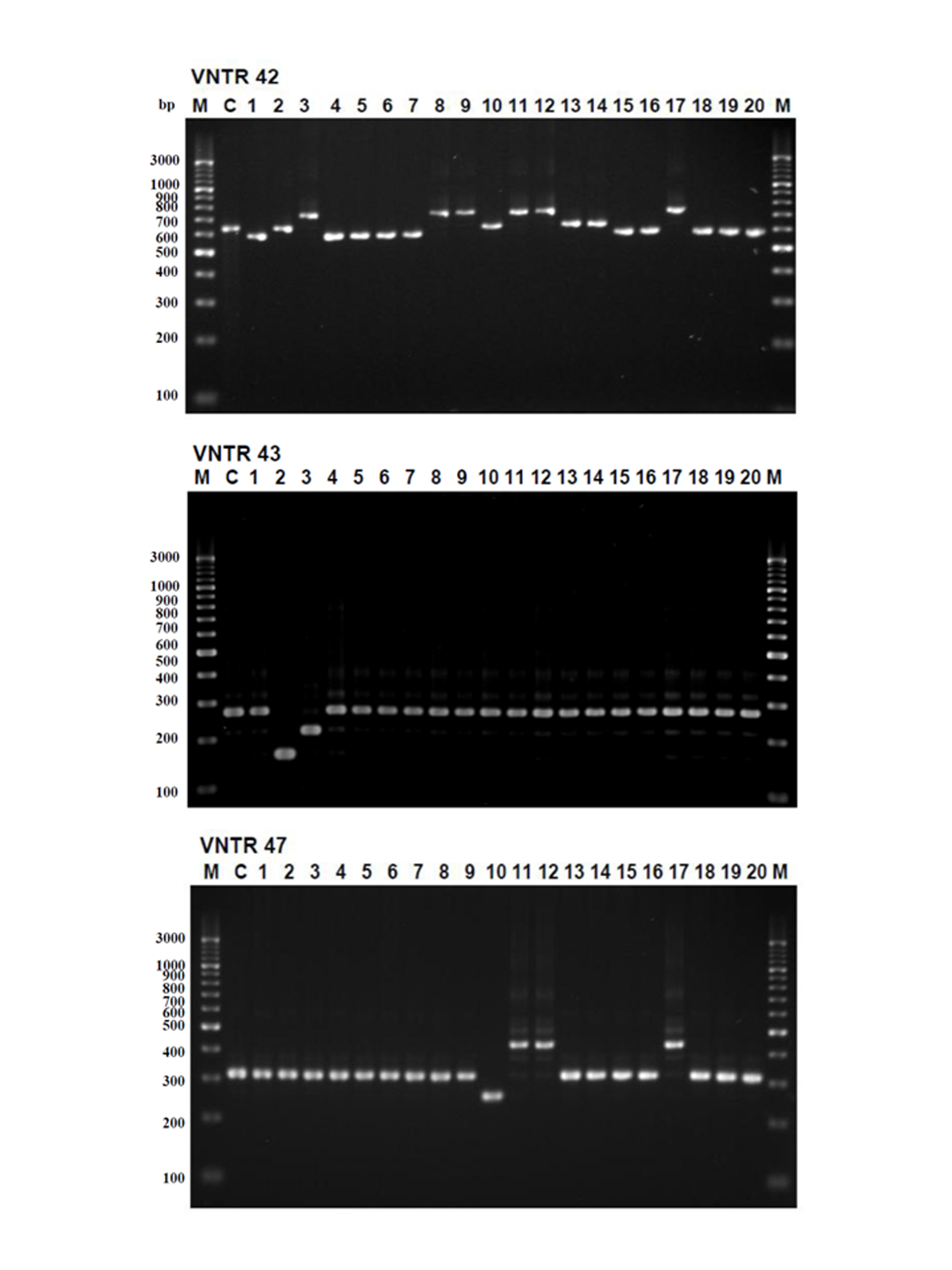

Supplement: Figure S1 — The quality test of MIRU-VNTR typing. Three loci was amplified and analyzed by electrophoresis using a 2% agarose gel. M, size markers; C, H37Rv control; lane 1–20; clinical isolates. (TIF) [file pone.0043018.s001.tif]
